# Asthma in a Primary Health Care Center Serving a Poor Population: A Descriptive and Interventional Study

**DOI:** 10.1097/WOX.0b013e31818a54c8

**Published:** 2008-12-15

**Authors:** Eloisa Malbrán, Graciela Laura Rey, Alejandro Malbrán

**Affiliations:** 1 Unidad de Asma, Alergia e Inmunología Clínica, Av. Roque Saenz Peña 1160 1 B, Capital Federal, Argentina

**Keywords:** asthma, minorities, health care system, accessibility, treatment, primary health care center

## Abstract

Asthma is a common chronic disease. Due to difficulties in accessibility to the health care system, asthma affects severely to minorities. This study's objective is to describe the morbidity of asthma on a poor population and its modification after abolishing assistance barriers.

Thirty-four asthma camps were carried out between May 2004 and May 2007. Patients' socioeconomic conditions, asthma history, and symptoms in the last month were determined during the camps. Patients received free medication and were invited to come to follow-up. Fifty-six children younger than 12 years old and 53 adults with persistent asthma were evaluated in 783 visits. The mean monthly income per capita was US $28.57.

At baseline, 50% of children and 34.5% of adults received inhaled corticosteroids. After intervention, 92.7% children and 98.1% adults received inhaled corticosteroids. Treatment was associated with a significant reduction of daytime and nighttime symptoms, absences to school or work, and emergency room visits and admissions. Patients referred less interference and more control of their disease in their everyday life.

Our results suggest that this population receives an insufficient treatment of its asthma severity. For such population, moving specialized assistance to the primary health care center resulted in a better control of their illness.

## 

Asthma is a common chronic illness; an estimated 5.8%[1] of people currently have asthma. Despite advances in diagnosis and treatment, asthma-associated morbidity and mortality have increased dramatically in the last 15 years, especially in poor and minority populations [2-6]. It is estimated, that asthma caused more than 1,800,000 visits to emergency departments, 500,000 hospitalizations, and more than 4000 deaths in the year 2004 in the United States [7]. Current treatment guidelines provide a framework to improve daily asthma control, prevent severe disease exacerbation, and diminish emergency visits and hospitalizations [8]. Decreasing asthma morbidity and mortality would reduce absences to school and work and health care resource utilization. Asthma severity seems to affect more severely poor and minority populations given elevated environmental and social exposures and suboptimal medical care [9].

In Argentina, 47.3% of the population is poor and 48.1% does not have health insurance and depends on public health care centers [10]. Public health service is organized in different levels of complexity: from the primary health center, run by municipal governments, where neighbors have access to medical care for common and prevalent diseases, through small local hospitals run by provinces for further diagnosis to highly sophisticated federal or university institutions, to treat more complex medical problems. All 3 levels are managed by different authorities, and there seems to be little communication among them. Primary health care centers are run by a family physician, a pediatrician, a gynecologist, a psychologist, and nurses. Its purpose is to bring medical assistance to the neighbor population, to decrease patients' medical access difficulties, and to diminish hospital muddle. In those cases in which the primary health center is not qualified to assist a patient, the patient is referred to the local general hospital [11]. However, poor and minorities have low access to the next level of care at the local hospital. Furthermore, in our area, free medication is provided by the primary health center but not by the hospital, and because poor patients cannot buy their own remedies, they rely for diagnosis on the hospital and on the primary health center for treatment.

According to asthma guidelines,[8] patients with moderate to severe symptoms must consult to a higher level of medical complexity and bronchial anti-inflammatory drugs should be used. However, due to the previously enunciated and other unknown reasons, poor and minority populations do not follow the predicted flow of medical assistance. Because dedicated asthma centers improve the quality of care and resource utilization for pediatric asthma in poor and minority populations,[12] we conducted this study (i) to describe the actual morbidity and treatment of asthma in a group of patients assisted in a primary health care center serving to a very poor population and ii) to describe the benefits of reducing financial and logistical barriers to care by bringing together the asthma specialist with free medication in the primary health center.

## Methods

### Primary Health Center

**Figure 1:**
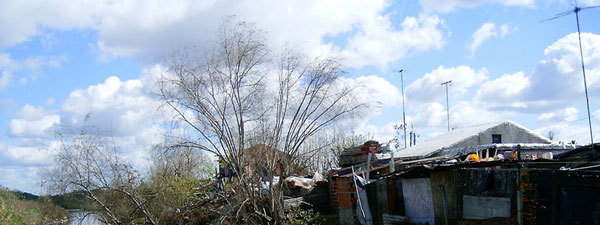
**Photograph of the area where the population lives**.

San Fernando is a suburban neighborhood of Buenos Aires bearing a very poor population. In accordance with the local authority, we located the Primary Health Center "Itallo Piaggi" to conduct this study. This center assists a low socioeconomic urban community, mostly living in crowded condition with a soil floor and without sanitation (Figure 1). The monthly per capita and per month income was calculated adding the income of all working members of each house divided by the total individuals living in it.

### Asthma Camps

Posters and local physicians informed asthma patients about asthma camps. Patients that considered themselves as asthmatics of any age or sex with or without a previously asthma diagnosis were enrolled and encouraged to participate. Asthma camps were settled once a month in a Saturday morning to prevent interference with working hours. A group of 1 or 2 Allergy and Immunology physicians, 3 medical students, and 2 volunteers took part in the camps. Patients were appointed at 9 AM. Upon their arrival, each patient fulfilled a questionnaire concerning (past) asthma history, disease symptoms in the last 4 weeks, family economy, and living conditions (see below). After fulfilling the questionnaire, patients were evaluated by a physician, properly diagnosed as asthmatics, classified according to guidelines into intermittent or mild, moderate or severe persistent disease and treated accordingly. Peak flow meters were not available for distribution. All patients with persistent asthma were invited to monthly follow-up.

Lectures on asthma were given by students to patients and their families. They consisted of a slide presentation explaining, in a simple and understandable way, the physiopathology of asthma and how to avoid triggers, to recognize exacerbations, and to treat them correctly.

#### Questionnaire

Adults and children older than 12 years old received a different questionnaire than children younger than 12 years old, mainly to account for cough as an asthma symptom [4]. Questionnaires for children were fulfilled by their parents or guardians who were required to accompany their kids to take part of the camp. Questionnaires addressed demographic, socioeconomic, and asthma information in 3 different sections: (i) demography, which included age, sex, race, height, weight, and level of education; (ii) socioeconomic level, which referred to total monthly income per house, adding the income of all workers living under the same roof; and (iii) asthma questions, which were organized in 3 groups. Group 1 referred to epidemiology: age at presentation, family history, previous evaluation by an allergist or a pneumonologist, and smoking status of the patient or the family. Group 2 referred to asthma history: last year school or work losses, admissions, and emergency visits. Finally, the group 3 included the last month course of illness: day and night frequency of symptoms, exercise induced asthma, number of days of school or work lost for asthma, admissions, emergency visits, physician visits, overall rate of symptom control by a 5-point scale from none to total control), limitations to work for asthma by a 5-step verbal descriptor from never to very often, dose and type of current medication, and other relevant pathological conditions. Asthma severity classification was established according to symptoms in the last month.

Although the primary objective of this study was to describe asthma symptoms in a poor population, a random group of patients was openly asked to state why they choose to continue treatment at the asthma camps instead of at the hospital. Answers were grouped in broad categories alike.

#### Doctor Interview

Once the questionnaire was fulfilled, patients were examined by the physician. Patients were requestioned about their asthma history and were physically evaluated. Asthma current severity was determined. Treatment was adjusted according to their illness severity step, and free needed drugs were given. Mild persistent, moderate, and severe asthma patients were asked to come to visit next month. At each visit, compliance with the management plan was established by asking the patients to bring back empty and partially used medication.

#### Asthma Intervention

Results of asthma intervention were assessed comparing indicators on asthma morbidity at baseline visit, at first follow-up (cross-time evaluation,) and as a mean of all follow-ups performed during the 3 years of the study, excluding the first (continuous evaluation). Variables analyzed included daytime symptoms, nighttime symptoms, frequency of rescue medication use, number of days missed from school or work, unscheduled visits to a physician, emergency visits, and hospital admissions.

#### Other tests

When appropriately, spirometry, before and after bronchodilators, was done with a Multispiro LT system (Irvine, CA).

#### Medication

Drugs prescribed were delivered at the asthma camp. Students trained patients and families to actuate inhalers correctly.

## Results

Thirty-four asthma camps were done between May 2004 and May 2007. A total of 783 visits were recorded. After the sixth camp, no more patients were incorporated due to medicine shortage. Results were analyzed in 2 groups: children up to 12 years old and adults (12 years and older).

### Demography

Ages ranged from 5 months to 83 years. Sixty-three percent of children were younger than 12 years old, and 74.6% of adults were Hispanic. Mean income per capita and per month was US $28.57. Camp statistics and demography results are detailed in Table 1.

**Table 1:** Camps Statistics and Patient Demography

	Adults and Children > 12	Children < 12	Both Groups
Total visits	287	496	783
First visits	85	115	200
Follow-up visits	202	381	583
Race, %			
Hispanic	74.60	62.30	70.90
White	23.80	37.7	27.90
Black	1.60	0	1.20
Age, mean ± SD (range)	39.5 ± 19.2 (12-83)	5.3 ± 3.2 (0.5-12)	-
Sex (F/M), %	77.6/22.4	35.7/64.3	5.25/4.75
Income, mean ± SD, US $ per month	32.09 ± 14.6	25.05 ± 13.4	28.57 ± 14
Members of the family, mean ± SD	4.8 ± 2.3	6.5 ± 2.9	5.7 ± 2.6

### Asthma History

Age at onset of asthma was at 21 ± 16.2 years (range, 0.1-64 years) in the adult group and at 1.7 ± 2.3 years (range, 0-9 years) in the children group. Thirty-one percent had a family history of asthma (mother, father, or both), being the mother in most cases (55.2%). Only 28.6% of patients had visited an asthma specialist before consulting to asthma camps, 32.6% of adults were current or past smokers, and 67.3% of children had at least 1 smoker member in their house.

### Children Younger Than 12 Years Old

Asthma class was defined according to the information collected as last month course of illness. One hundred fifteen children were evaluated. Nine did not have asthma and 15 were classified as mild intermittent asthma and were referred to their primary care provider for follow-up.

Thirty-five patients had wheezing episodes associated with upper respiratory infections but were asymptomatic the rest of the time. This group was 3.8 ± 2.6 years old, significantly younger than persistent asthmatic children (5.3 ± 3.3 years; *P *< 0.03), 25% were on inhaled steroids at arrival and 37% after intervention. This group was treated according to risk stratification[4] and was not included in the asthma impairment analysis.

Fifty-six patients were diagnosed as persistent asthmatics, 22 (39.3%) had mild, 31 (55.4%) had moderate, and 3 (5.4%) had severe persistent asthma and were offered treatment under specialist care. According to asthma class at arrival, 100% of patients had indication of inhaled steroids (mild + moderate asthma + severe persistent asthma) but only 50% received them, 17.9% relied on albuterol, 7.1% were on oral steroids, and 25% had no treatment. After asthma camp intervention, 92.7% of patients received inhaled steroids treatment, 29.1% of them combined with long-acting B_2 _agonists.

Thirty patients (53.5%) returned to the camp for follow-up after 37.6 ± 19.9 days (range, 14-105 days). Severity of asthma at second camp was similar to the whole group at first camp: 13 (43.3%) were classified as mild, 15 (50%) as moderate, and 2 (6.7%) as severe asthmatics. Cross-sectional evaluation of the benefits of treatment intervention was analyzed comparing last month course of illness at baseline and at first follow-up. Continuous benefits of treatment on the course of the disease were demonstrated obtaining the average of the results of all controls, excluding first visit and first follow-up. Day and nighttime symptoms per week during the previous month as well as rescue medication use diminished significantly from baseline to first follow-up and continued that way through all camps (Table 2). Improvement in asthma control lowered absences to school, emergency room (ER) visits, and unscheduled visits. Incidence and frequency of exercise induced asthma were reduced, and patients were less frustrated coping with their disease (data not shown). According to the age of this group, improvement in the quality of life involved both the patient and its family (Table 3).

**Table 2:** Asthma-Related Symptoms in Children

	Total Patients, Baseline	First Follow-up	Average Next Follow-ups
Daytime symptoms per week	3 ± 2.6	1.3 ± 1.8*	0.8 ± 1.6
Nighttime symptoms per week	2.8 ± 2.5	1.6 ± 2.2**	0.7 ± 1.6
Cough days per week	4.1 ± 2.4	3.1 ± 2.7, NS	1.8 ± 2.4
Use of daytime rescue medication per week	3.7 ± 2.9	1.5 ± 1.8^‡^	0.9 ± 1.7
Use of nighttime rescue medication per week	3.1 ± 2.8	0.9 ± 1.5^‡^	0.6 ± 1.4

*P < 0.05

**P < 0.01

^‡^*P *< 0.001

NS indicates not significant.

**Table 3:** Asthma Morbidity in Children

	**Total Patients**, Baseline	First Follow-up	Average next Follow-ups
School absences (yes/no), n	27/17 (44)	7/15* (22)	43/213* (256)
ER visits (yes/no), n	23/22 (45)	9/16 (25)	32/281** (313)
Doctor visit without appointment (yes/no), n	25/20 (45)	4/20** (24)	24/287** (311)
Do these symptoms interfere in your physical activity?			
Never, n (%)	11 (22.9%)	11 (47.8%)	189 (64.5%)
Almost never, n (%)	1 (2.1%)	2 (8.7%)	19 (6.5%)
Sometimes, n (%)	15 (31.3%)	2 (8.7%)	48 (16.4%)
Often, n (%)	10 (20.8%)	4 (17.4%)	20 (6.8%)
Very often, n (%)	11 (22.9%)	4 (17.4%)	17 (5.8%)
How controlled are your symptoms?			
Nothing, n (%)	4 (19%)	0 (0%)	3 (3.1%)
Little, n (%)	6 (28.6%)	1 (7.1%)	21 (7.3%)
Some, n (%)	4 (19%)	2 (14.3%)	41 (14.2%)
Good, n (%)	5 (23.8%)	7 (50%)	143 (49.5%)
Totally, n (%)	2 (9.5%)	4 (28.6%)	81 (28%)

*P < 0.05

**P < 0.01

### Adults and Children Older Than 12 Years Old

Asthma class was defined according to the course of the illness in the last month, recorded during the first visit. Eighty-six adults were evaluated. Eleven patients did not have asthma, 5 had chronic obstructive pulmonary disease, 5 had a present asthmatic exacerbation, and 12 were classified as mild intermittent asthma and were referred to their primary care provider for follow-up. Fifty-three patients were diagnosed as persistent asthmatics, 5 (9.4%) had mild, 39 (73.6%) had moderate, and 9 (17%) had severe persistent asthma and were offered treatment under specialist care. According to asthma class at arrival, 100% of patients had indication of inhaled steroids (mild + moderate + severe persistent asthma) but only 34.5% received them, 36.2% relied on albuterol, 3.4% were on theophylline, and 25.9% had no treatment. After asthma camp intervention, 98.1% of patients received inhaled steroids treatment, 83% of them were combined with long-acting B_2 _agonists.

Twenty-six patients (49%) returned to the camp for follow-up after 45.2 ± 32.3 days (range, 14-126 days). This group included 3 patients (11.5%) with mild asthma, 18 patients (69.2%) with moderate asthma, and 5 patients (19.2%) with severe asthma. Benefits of treatment intervention were analyzed with the same strategy than for children. Significant symptoms improvement and asthma control were recorded from baseline to first follow-up; the improvement remained stable across the study and continued that way through all camps (Table 4). Patients reported a reduction in the number of absences to job, admissions, visits to ER, and physician visits without an appointment, and patients were less frustrated coping with disease (Table 4)

**Table 4:** Asthma-Related Symptoms in Adults

	Total **Patients--Baseline**, N = 58	First **Follow-up**, N = 27	Average Next **Follow-ups**, N = 190
Daytime symptoms per week, mean ± SD	4.2 ± 2.8	3.2 ± 2.8	1.6 ± 2.2
Nighttime symptoms per week, mean ± SD	3.7 ± 2.6	2 ± 2.5	1.3 ± 2.1
Work absences (yes/no), n	16/26 (42)	4/16 (20)	7/81* (88)
Admissions (yes/no), n	9/49 (58)	0/27** (27)	'0/189** (189)
Visits to ER (yes/no), n	27/31 (58)	7/20 (27)	13/177* (190)
Doctor visits without appointment (yes/no), n	16/42 (58)	5/22 (27)	11/178* (189)
Do these symptoms interfere in your working capacity?			
Never, n (%)	8 (15.7%)	9 (37.5%)	80 (51.3%)
Almost never, n (%)	4 (7.8%)	1 (4.2%)	7 (4.5%)
Sometimes, n (%)	8 (15.7%)	5 (20.8%)	34 (21.8%)
Often, n (%)	10 (19.6%)	2 (8.3%)	8 (5.1%)
Very often, n (%)	21 (41.2%)	7 (29.2%)	27 (17.3%)
How controlled are your symptoms?			
Nothing, n (%)	14 (25.9%)	1 (3.7%)	2 (1.1%)
Little, n (%)	13 (24.1%)	3 (11.1%)	16 (8.7%)
Some, n (%)	17 (31.5%)	10 (37%)	41 (22.4%)
Good, n (%)	9 (16.7%)	9 (33.3%)	106 (57.9%)
Totally, n (%)	1 (1.9%)	4 (14.8%)	18 (9.8%)

*P < 0.001

**P < 0.03

### Why Patients Come to Asthma Camps But Do Not Go to the General Hospital?

Eighteen patients or their tutors answered this question. Answers were grouped in 3 broad categories: (i) good quality of care, 8; (ii) accessibility, 8; and (iii) free medication, 2.

## Discussion

Long-term studies of asthma care in a community setting are critically important for the implementation of effective and evidence-based health care services, particularly for lower socioeconomic populations that experience high rates of asthma morbidity [12]. These patients are often treated in emergency and urgent care facilities for exacerbation; remaining with an inadequate preventive maintenance strategies and an increased risk of exacerbations, frequent unscheduled visits for care, and dangerous overuse of rescue medications [6,13,14]. There have not been studies addressing the asthma status of poor populations in Argentina. We decided to prospectively investigate the actual status of asthma control in the poor and its modification by adequate treatment. The 109 persistent asthmatics included in this study were assisted at the Italo Piaggi Primary Health Center, devoted to care for a very poor population near Buenos Aires. They were selected out from a group of 200 patients that defined themselves as asthmatics and voluntarily came to asthma camps for evaluation and treatment. Patients belong to a very poor population living in crowded conditions with a mean income well below poverty line. At their initial evaluation, this selected group of patients was grossly undertreated and undereducated about their illness, and their disease had a very negative impact on productivity and quality of life. This initial evaluation reproduces other similar studies of asthma in poor and minority people [5,15].

Patients were previously treated by general or family physicians who run the primary health centers. Although the system is organized in such way that persistent asthma patients can assist to the general hospital for evaluation and treatment, it fails. Most of our patients failed to seek specialized care in their local hospital. Only 28.6% of them were evaluated by an asthma specialist although they have persistent disease and free of charge access to the local hospital. Patients remain under the general physician care. This situation leads to an inadequate treatment, more asthma complications, a poor quality of life, and an increase in cost for society as a whole [5]. Components of health care access include the ability to get into the health care system as well as to obtain appropriate care once in the system. The availability of health care providers who meet an individual patient's needs is another key component of access to care. Poor populations' incapacity to access medical help has been documented, even when corrected by level of education and health insurance [16]. Our patients showed the same kind of limitations to access to health care.

The optimal management of asthma is frequently compromised by the lack of a comprehensive clinical management program. Multidimensional programs have not been widely instituted or tested in clinics serving low-income and ethnically diverse populations like ours. Ideally, such programs would emphasize multiple elements including, among others, use of evidence-based clinical practice guidelines, use of in situ simple diagnostic procedures such as spirometry, patient, and family education, and support of disease self-management programs. The results of the study confirmed our hypothesis: moving the asthma specialist to the primary health center and training patients and family in disease management would by-pass access to care barriers and reduce asthma morbidity. The findings obtained in the cross-sectional analysis at follow-up disclosed a very significant improvement in all areas of asthma control, from symptoms scores to quality-of-life measurements. Furthermore, the continuous analysis of average severity of asthma scores per patient during the 3 years of study showed a persistent benefit for patients adhering to the camps. This is shown in the previous tables, which express a significant drop in symptoms, absences to school and work, visits to physicians or ER, and hospitalizations. Unfortunately, only half of persistent asthmatics diagnosed at the first consult continued into the study. We did not attempt to locate those individuals not returning to the asthma camp when expected. We believe that using a system to incentivize adherence to care at the primary heath center will increase the number of patients returning for follow-up.

Although the primary objective of our study was to document actual asthma control and improvement with a structured approach, we questioned patients about their ultimate reason for coming to the camp for continuous treatment. As expected, 62% of answers could be grouped as better access to care: closeness to residence, little waiting time, and provision of medication as the main reason for their coming. However, 38% referred better quality of care as the main value of the camps reflecting patients' frustration in their attempt to get appropriate medical attention in a system too complex for their ability to access care.

We conclude that poor populations have a suboptimal medication and large asthma-related morbidity. This could be improved by moving the asthma specialist to the primary health center and by reversing the actual flow of medical attention, resulting in better asthma outcomes. Hence, the possibility of taking the specialist near this population must be considered.

## Notes

Sources of support: self funded.

## References

[B1] Eder W Ege MJ Von Mutius E The asthma epidemia N Engl J Med 2006 1 2226 2235 10.1056/NEJMra054308 17124020

[B2] Kattan M Mitchell H Eggleston P Gergen P Crain E Redline S Characteristics of inner-city children with asthma: The National Cooperative Inner-City Asthma Study Pediatr Pulmonol 1998 1 4 253 262 10.1002/(sici)1099-0496(199710)24:4<253::aid-ppul4>3.0.co;2-l 9368259

[B3] Halterman JS Aligne CA Auinger P McBride JT Szilagyi PG Health and health care for high-risk children and adolescents: inadequate therapy for asthma among children in the United States Pediatrics 2000 1 1 272 276 10617735

[B4] Landon BE Hicks LS O'Malley J Lieu TA Keegan T McNeil BJ Improving the management of chronic disease at community health centers N Engl J Med 2007 1 921 934 10.1056/NEJMsa062860 17329699

[B5] Levy JI Welker-Hood LK Clougherty JE Dodson RE Steinbach S Hynes HP Lung function, asthma symptoms, and quality of life for children in public housing in Boston: a case-series analysis Environ Health 2004 1 13 25 10.1186/1476-069X-3-13 15585065 PMC544563

[B6] Fox P Porter PG Lob SH Holloman Boer J Rocha DA Adelson JW Improving asthma-related health outcomes among low-income, multiethnic, school-aged children: results of a demonstration project that combined continuous quality improvement and community health worker strategies Pediatrics 2007 1 e902 e911 10.1542/peds.2006-1805 17908746

[B7] Akinbami L Asthma Prevalence, Health Care Use and Mortality: United States, 2003-05 2008 Washington, (DC): Office of Analysis and Epidemiology, Centers for Disease Control and Prevention, US Department of Health and Human Services

[B8] National Asthma Education and Prevention Program Expert Panel Report 3: Guidelines for the Diagnosis and Management of Asthma Full Report 2007. National Heart, Lung, and Blood Institute. National Institutes of Health NIH Publication No. 07-4051

[B9] Rona RJ Asthma and poverty Thorax 2000 1 239 244 10.1136/thorax.55.3.239 10679545 PMC1745704

[B10] Indicadores de recursos, acceso y cobertura--2003--Dirección de estadísticas y recursos de la salud. Ministerio de Salud. Presidencia de la Nación República Argentina

[B11] Lemus JD Salud publica, epidemiología y atención primaria de salud 2005 CIDES Argentina

[B12] Jones CA Clement LT Morphew T Kwong LY Hanley-Lopez J Lifson F Achieving and maintaining asthma control in an urban pediatric disease management program: The Breathmobile Program J Allergy Clin Immunol 2007 1 6 1445 1461 10.1016/j.jaci.2007.02.031 17416407

[B13] Diette GB Skinner EA Markson LE Algatt-Bergstrom P Nguyen TT Clark RD Consistency in care with national guidelines for children with asthma in managed care J Pediatr 2001 1 59 64 10.1067/mpd.2001.109600 11148513

[B14] Anis AH Lynd LD Wang X King G Spinelli JJ Fitzgerald M Double trouble: impact of inappropriate use of asthma medication on the use of health care resources CMAJ 2001 1 625 631 11258208 PMC80815

[B15] Karen L Warman KL Johnson Silver E Stein REK Asthma symptoms, morbidity, and antiinflammatory use in inner-city children Pediatrics 1 277 282 10.1542/peds.108.2.277 11483788

[B16] Goldman L Ausiello D Cecil Textbook of Medicine 2007 Saunders, Elsevier: Philadelphia, PA

